# Dutch taboo norms

**DOI:** 10.3758/s13428-017-0890-x

**Published:** 2017-04-13

**Authors:** Sander A. Roest, Tessa A. Visser, René Zeelenberg

**Affiliations:** 1 Department of Psychology, Erasmus University Rotterdam, Rotterdam, The Netherlands; 2 Department of Psychology, Erasmus University Rotterdam, Woudestein T16-03, P.O. Box 1738, 3000 DR Rotterdam, The Netherlands

**Keywords:** Lexical norms, Taboo ratings, Valence ratings, Arousal ratings, Emotion

## Abstract

This article provides norms for general taboo, personal taboo, insult, valence, and arousal for 672 Dutch words, including 202 taboo words. Norms were collected using a 7-point Likert scale and based on ratings by psychology students from the Erasmus University Rotterdam in The Netherlands. The sample consisted of 87 psychology students (58 females, 29 males). We obtained high reliability based on split-half analyses. Our norms show high correlations with arousal and valence ratings collected by another Dutch word-norms study (Moors et al.,, *Behavior Research Methods, 45,* 169–177, 2013). Our results show that the previously found quadratic relation (i.e., U-shaped pattern) between valence and arousal also holds when only taboo words are considered. Additionally, words rated high on taboo tended to be rated low on valence, but some words related to sex rated high on both taboo and valence. Words that rated high on taboo rated high on insult, again with the exception of words related to sex many of which rated low on insult. Finally, words rated high on taboo and insult rated high on arousal. The Dutch Taboo Norms (DTN) database is a useful tool for researchers interested in the effects of taboo words on cognitive processing. The data associated with this paper can be accessed via the Open Science Framework (https://osf.io/vk782/).

Emotional word norms are available in many different languages (e.g., Bradley & Lang, 1999; Moors et al., 2013; Monnier & Syssau, 2014; Warriner, Kuperman, & Brysbaert, 2013). These norms usually contain ratings on arousal, valence, and dominance. Recently, Janschewitz (2008) compiled a normative list of the tabooness of a large number of English words. These taboo words include profanities, vulgarities, sexual terms, racial slurs, and insults (see Jay, 2009, for a short review of the use of taboo words in psychological science). Taboo words have been widely used in psychological research (e.g., Buchanan, Etzel, Adolphs, & Tranel, 2006; Jay, Harris, & King, 2008; Madan, Shafer, Chan & Singhal, 2017; Mathewson, Arnell, & Mansfield, 2008; Severens, Kühn, Hartsuiker, & Brass, 2012). To date no normative data about the tabooness of words has been collected in languages other than English. Because the tabooness of words is heavily influenced by social aspects (Jay, 2009), it is important to generate normative data about taboo words in different languages. We constructed a list of 672 Dutch words, including 202 taboo words, and asked participants to rate these on general taboo, personal taboo, insult, arousal, and valence. We compiled them into the Dutch Taboo Norms (DTN).

Taboo words are words that *“are sanctioned or restricted on both institutional and individual levels under the assumption that some harm will occur if a taboo word is spoken”* (Jay, 2009). As such, people may be hesitant to use taboo words amongst others. People learn what words are inappropriate to say and in what situations through socialization. What words are inappropriate and to what extent may depend on the context in which they are used. Certain words might be fine to use when quipping among friends but might be highly inappropriate when you are in the same room as your parents. Because of this context-dependency, it is difficult to pinpoint which words are taboo or when they are considered taboo. Individual differences, for example in religious conviction and gender, may also influence which words are considered inappropriate. Because of these differences, there may be a difference between general and personal tabooness. In addition, some words might be taboo but rarely used as an insult (e.g., *orgy* or *blowjob*). It is therefore important to be able to differentiate between insulting and not insulting taboo words. To summarize, the use and evaluation of taboo words is probably affected by several contextual factors.

Males and females, for example, differ in the specific way and frequency with which they use taboo words (e.g., Bailey & Timm, 1976; McEnery & Xiao, 2004; Thelwall, 2008). Men tend to swear more frequently than women do and men and women seem to differ slightly in what type of swear words they use (e.g., men use fuck more frequently than women while women use bitch more frequently than men). The Janschewitz (2008) taboo study shows gender differences; men yielded higher ratings on personal use, arousal, and imageability for taboo words than women did.

Besides differences in gender, Janschewitz (2008) found that religious participants rated taboo words as more offensive than non-religious participants did. It could be that religious people are more sensitive to taboo words. For example, strict Christians show a neural response to statements that clash with their value system (e.g. “I think euthanasia is an acceptable course of action”) while non-Christians with an opposing value system show a neural response to the opposing statement (Van Berkum, Holleman, Nieuwland, Otten, & Murre, 2009). This suggests that certain words or statements are processed differently by religious people than by nonreligious people. It is therefore important to add information about religiosity to the DTN.

﻿Some researchers see taboo words as a separate class of emotional words (e.g., Jay, 2009; Janschewitz, 2008). For example, taboo words are remembered better than other words even when compared to semantically related low arousal words or emotional words (e.g., Buchanan et al., 2006). Taboo words also attract more attention than emotional words in rapid serial visual presentation (RSVP) tasks (Mathewson et al., 2008) and people need more time to process taboo words than neutral words (Dorfman, Grossberg, & Kroeker, 1965; see also, Erdelyi, 1974). In a recent study, taboo words were associated with slower response times in lexical decision than emotional words and neutral words when controlling for non-emotional word properties (Madan, Shafer, Chan & Singhal, 2017). There have been a few studies that used Dutch taboo words as stimuli (e.g., Severens et al., 2012; Zeelenberg, Bocanegra, & Pecher, 2011). These studies collected their own data on a small set of possible taboo words and did not make these norms public. To study the effects of taboo words on cognitive processes it is useful to have normative data about the tabooness of words that is easily available so that researchers do not have to collect their own norms for each individual study.

Moors et al. (2013) collected affective norms for Dutch words. This study included 4,300 words and has ratings for valence, arousal, and dominance. Ratings were given on 7-point Likert scales. Although this database includes a number of words that can be considered taboo words, Moors et al. did not include taboo ratings. Moreover, many words that could be considered taboo are not included in these norms.

The aim of the current study is to provide researchers with information regarding the tabooness and level of insult of a number of Dutch words. We chose a sample of psychology students because this is the population often tested in studies on emotion. The study includes affectiveness ratings (valence and arousal), general taboo ratings, personal taboo ratings, and insult ratings of 672 Dutch words, including 202 taboo words. All participants rated the words on general taboo, personal taboo, and insult. Half the participants completed a valence rating and the other half completed an arousal rating in addition to the other variables. This was done so that participants took less time to complete the list and to eliminate the possibility of arousal and valence ratings influencing each other. Participants were also asked to think about the context in which the word could be taboo or insulting and write down this context for each word. We hoped that these contexts would provide additional information regarding the use and interpretation of taboo words.

## Method

### Participants

One hundred psychology students at the Erasmus University Rotterdam participated in the experiment (68 females, 32 males). Ten participants (eight females, two males) were removed because they indicated they were not native speakers of Dutch. Three participants (two females, one male) were removed because of a high number of outlying scores and/or because the ratings had been seemingly entered at random. The norms presented here reflect the data of the 87 remaining participants. Participants received course credit for participating in the study. Participants were randomly assigned to rate either the valence or arousal of words in addition to tabooness and insult. 1

### Materials

Six hundred and seventy-two words were included in this study: 202 taboo words, 60 positive valence–low arousal words, 60 positive valence high–arousal words, 60 negative valence–low arousal words, 60 negative valence–high arousal words, and 230 emotionally neutral words. Similar to Janschewitz (2008), taboo words were chosen according to the experimenters’ discretion and included words related to sexuality (e.g., *vagina [vagina]*, *porno [porn]*); scatology (e.g., *stront[shit]*, *pis[piss]*); racial slurs (e.g., *neger[negro]*, *allochtoon[first or second generation immigrant]*); religion (e.g., *Godverdomme [Goddammit]*, *Jezus[Jesus]*); and other insults (e.g., *imbeciel [imbecile]*, *hufter[asshole]*). We also added diseases (e.g., *kanker[cancer]*, *tering[tuberculosis]*), and compounds (e.g., *kankerhoer[cancer whore]*, *teringlul[tuberculosis dick]*)^2^ because these combinations might elicit even higher taboo ratings than single taboo words that are not combined in compounds. Of the 202 taboo words, 59 were included in the database compiled by Moors et al. (2013).

The 240 affective words in our study were selected from Moors et al. (2013). They compiled a database consisting of 4,300 Dutch words that have been rated on arousal, valence, and dominance. Ratings were given on a 7-point Likert scale. We selected 240 words from the study of Moors et al. based on the arousal and valence ratings. Those words were selected to create four different sets; positive valence words of low or high arousal and negative valence words of low or high arousal.

In addition, we selected 230 neutral words from different categories from De Deyne et al. (2008). We selected 26 birds, 25 clothing related words, 18 fruits, 26 kitchen related words, 20 living-room related words, 23 mammals, 24 sports, 25 tools, 20 vegetables, and 23 vehicles. Of these 230 words, 131 words featured in Moors et al. (2013). Table 1 shows the means and standard deviations of the valence and arousal ratings that Moors et al. obtained for all words that were also presented in the current study.

**Table 1:** Means and standard deviations (in parentheses) for valence and arousal ratings for taboo, positive, negative, and neutral words (Moors et al., 2013)

Word type	Number of words in common	Valence	Arousal
Taboo	59	3.24 (1.35)	4.66 (0.91)
Positive			
High arousal	60	5.95 (0.21)	5.49 (0.29)
Low arousal	60	5.32 (0.28)	3.02 (0.40)
Negative			
High arousal	60	1.89 (0.24)	5.56 (0.39)
Low arousal	60	2.40 (0.35)	2.57 (0.31)
Neutral	131	4.30 (0.43)	3.96 (0.78)

*Note*. Number of words in common refers to the number of words for each word type that was rated in both the Moors et al. (2013) study and the present study. Ratings for positive and negative words are broken down by arousal level (high and low)

### Procedure

Each participant rated the entire set of 672 words on either arousal or valence, as well as on general tabooness, personal tabooness, and insult. Participants were also asked to provide a context in which they believe the word might be taboo or insulting.

Moors et al. (2013) collected data by sending participants an Excel file containing all 4,300 words. Participants were asked to rate all words on one of the variables and to send the filled in forms back via email. In our study, participants entered ratings in an Excel file similar to the procedure used by Moors et al. (2013). The Excel file contained two sheets: The first sheet contained instructions and the second sheet contained the 672 words. Participants who had signed up for the study received this file via email and were asked to complete the ratings. They received either a version in which they had to rate words on arousal or a version in which they had to rate words on valence, in addition to general taboo, personal taboo, and insult ratings. To randomize the word order for each list we used the following method: using Excel, we created 55 lists with randomized word order, each of these 55 lists was used for both the arousal and the valence rating. In total we made 110 different files.^3^ Examples of both a valence and an arousal version are provided as supplementary materials on OSF (https://osf.io/vk782/).

Participants were instructed to rate the words on a 7-point Likert scale. The first sheet of the Excel file contained explanations regarding these ratings. Table 2 shows the labels used for each rating score and variable. Context was not rated; rather we asked participants to name a context in which the word might be taboo or insulting.

**Table 2:** Labels used for ratings scores for each variable

Rating score	Rating variables			
	Valence	Arousal	Taboo^a^	Insult
1	Very negative	Very passive	Not taboo	Not insulting
2	Fairly negative	Fairly passive	Very little taboo	Very little insulting
3	Somewhat negative	Somewhat passive	Little taboo	Little insulting
4	Neutral	Neutral	Moderately taboo	Moderately insulting
5	Somewhat positive	Somewhat active	Somewhat taboo	Somewhat insulting
6	Fairly positive	Fairly active	Fairly taboo	Fairly insulting
7	Very positive	Very active	Very taboo	Very insulting

a. The same labels were used for the General Taboo and Personal Taboo Rating Scales

The rating sheet consisted of ten columns in an Excel file that was sent to participants by email. The first column contained the words that participants had to rate. The cells in the second column were initially empty. Participants used this column to enter their rating for valence or arousal (depending on the version to which they were assigned). The cell in the third column was empty until the corresponding cell in the second column was filled in, at which time the verbal label (e.g., very active/aroused) corresponding to the number entered in the second column (e.g., 7) appeared. In a similar fashion, the fourth and fifth columns were used for rating general tabooness, the sixth and seventh columns were used for rating personal tabooness, the eighth and ninth columns were used to rate insult, and the tenth column was used to fill in a possible context. The file was protected in such a way that participants could only fill in the blanks in columns two, four, six, and eight but could not change the word order or fill in the columns where the verbal labels would appear.

The entire experiment took about four hours to complete. Participants were not given a deadline to return the completed rating sheet, but were contacted by email a month after they had been sent the rating sheet if they had not completed and returned the ratings by that time. After that, they were given a time limit of 2 weeks to complete filling in the rating sheet. Thus, in total they were given about 6 weeks to complete the ratings. After they returned the completed rating sheet they received a short questionnaire via email that asked about their gender, age, native language, and religion. If they indicated being religious, they were asked how many times they visit their house of worship (1 = at least once a week, 2 = between once a week and once a month, 3 = less than once a month, 4 = never). Participants received course credit after they replied to this questionnaire.

## Results

### Data preprocessing

We first removed all ratings in which participants noted that they did not know the word. This was only the case on three occasions. Next, we removed all ratings that fell outside the 1–7 range which participants were required to use for their ratings. This was the case on 86 occasions (0.04% of all ratings). A few words featured twice in the rating list by accident (*anuslikker, muts, pot, sloerie, slachten, tyfusslet*). We decided to use the first rating of these words for further analyses and excluded the second rating. Next, we calculated the mean and standard deviation for all words for each variable. We then counted the number of ratings that were below or above the average by more than 2.5 standard deviations for each participant.

In total thirteen participants were removed. Ten participants were non-native Dutch speakers (eight females, two males). One participant (male) was removed because of a high number of outliers (33.8%) across all rating dimensions. Another participant (female) was removed because of a high number of outliers in arousal scores (41.6%). The final participant (female) that was removed had a lower number of outliers (11.7%), but after looking at the ratings of this participant, we noticed that most ratings seemed random. No other participants were removed.^4^

### Supplementary materials

Six files are provided as supplementary materials to this article (https://osf.io/vk782/). The raw data have been compiled and added to an Excel file containing overall means and standard deviations. Information for all participants is combined on the first sheet, females only on the second sheet, males only on the third sheet, religious participants only on the fourth sheet, and nonreligious participants only on the fifth sheet. The first sheet of the Excel file has been copied into an SPSS file and a.cvs file. This was done to make the use of DTN more convenient for researchers who prefer these formats. We have provided an example of a valence and an arousal version of the word lists as they were send to participants. Finally, we have provided an excel file that includes all the raw data collected in this study. The first sheet of this file contains demographic information about gender, native language, religiosity, and whether participants rated words on valence or on arousal. All other sheets contain the raw data on all variables, one participant per sheet.

The overall data files contain all 672 Dutch words in alphabetical order. English translations are given; these translations are based on Google Translate, http://www.mijnwoordenboek.nl, and the authors’ discretion. Some words did not have a clear translation equivalent in English. This was primarily the case for a number of taboo words, for which we added a literal translation (lit.) or approximate translation (approx.). Literal (i.e., word-for-word) translations (e.g., *cancer whore* for the Dutch word *kankerhoer*) were only given when we could not think of a good approximate translation. This file contains the *means (M)* and *standard deviations (SD)* for arousal, valence, general taboo, personal taboo, and insult ratings. In addition, we added information regarding word *Category* and word *Type*. The variable *Category* contains information on which category the word belongs to: *taboo*, positive valence–low arousal (*pos low arousal*), positive valence–high arousal (*pos high arousal*), negative valence–low arousal (*neg low arousal*), negative valence–high arousal (*neg high arousal*), *birds*, *clothing*, *fruit*, *kitchen*, *living room*, *mammal*, *sport*, *vegetable*, or *vehicle*. The variable *Type* contains more general information on word type: *Taboo*, *Positive*, *Negative*, or *Neutral*. For convenience we also added word *length*, number of *syllables*, frequency per million (*FreqPM)*, log10 per million (*Log10PM*), a logarithmic scale that standardizes log10 per million words (*Zipf*), and grammatical category (*GramCat*). Frequency per million words ratings were taken from the SUBTLEX-NL database (Keuleers, Brysbaert, & New, 2010). Log10 per million words were calculated using the frequency per million words. Zipf ratings were calculated based on the word frequency in SUBTLEX-NL, size of the SUBTLEX-NL corpus, and total number of individual words in the SUBTLEX-NL corpus (Van Heuven, Mandera, Keuleers, & Brysbaert, 2014). Fifty-six words in our study do not appear in SUBTLEX-NL, we therefore assigned the values of frequency per million = .02 and log10 frequency per million = -1.64 to these words (Brysbaert & New, 2009; Keuleers et al., 2010). Zipf values correct for words that do not appear in a corpus by adding a frequency of 1 to all words. *Grammatical category* contains information about which type each word mainly belongs to according to the SUBTLEX-NL database (some words might fall into multiple categories, depending on context). Each word is categorized as a noun (N), adjective or adverb (A), or verb (V).

### Reliability

We calculated three different measures of reliability. These include (1) split-half correlation analyses between even numbered and uneven numbered participants, (2) correlation analyses on general taboo, personal taboo, and insult ratings between participants who gave the arousal ratings and participants who gave valence ratings, and (3) correlations between our arousal and valence ratings and those collected by Moors et al. (2013).

In order to calculate the split-half correlation, we divided the dataset in two subsets based on participant number (even or uneven). For each subset, we then calculated the mean ratings for each word for each rating variable. Overall, this analysis shows high agreement between subsets on all rating variables; for arousal, *r* = .92, valence, *r* = .98, general taboo, *r* = .99, personal taboo, *r* = .98, and insult, *r* = 99.

In order to calculate the correlations for general taboo, personal taboo, and insult ratings between participants who rated arousal and participants who rated valence we calculated the means on each of these variables per group. This analysis showed high correlation between these groups; for general taboo, *r* = .99, personal taboo, *r* = .98, and insult, *r* = .99.

**Fig. 1:**
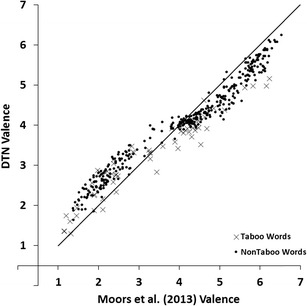
Mean valence ratings of the Dutch Taboo Norms (DTN) plotted against the mean valence by Moors et al. (2013). Lower scores indicate more negative ratings, higher scores indicate more positive ratings

**Fig. 2:**
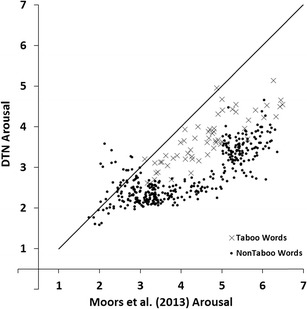
Mean arousal ratings of the Dutch Taboo Norms (DTN) plotted against the mean arousal by Moors et al. (2013). Lower scores indicate more passive ratings, higher scores indicate more active ratings

In order to compare our ratings with the Moors et al. (2013) database we calculated the correlations between our arousal and valence ratings, and those collected by Moors et al. The DTN contains 430 words that also featured in Moors et al. Figure 1 shows mean valence ratings of the DTN against mean valence ratings obtained by Moors et al. Figure 2 shows mean arousal ratings of the DTN against mean arousal ratings obtained by Moors et al. In all figures, taboo words are represented by a different symbol (i.e., ×) than non-taboo (positive, neutral and negative) words (i.e., ●). Valence showed a correlation of *r* = .98 and arousal showed a correlation of *r* = .77.

Visual inspection of Fig. 1 reveals that the mean valence scores in our study were somewhat less extreme than those in Moors et al. (2013). That is, words that received low valence ratings in Moors et al. received slightly higher ratings in our study and words that received high valence ratings in Moors et al. received slightly lower ratings in our study. This may reflect a general tendency of our participants to avoid extreme valence ratings. Alternatively, this may reflect a (small) regression to the mean effect. The negative and positive words that we selected from Moors et al. consisted of the more extremely rated words from that study. Consequently, one would expect the valence scores for these words to be less extreme in a new sample. Visual inspection of Fig. 2 reveals that most words received lower arousal ratings in our study than in Moors et al. In a similar vein, the taboo rating study of Janschewitz (2008) found lower arousal ratings for words that had also been rated in the ANEW study. Possible reasons for this observation are provided in the Discussion.

### Mean ratings

Table 3 shows the mean ratings for all words, positive and negative words broken down by arousal level (high and low, as they were selected from Moors et al., 2013). As expected, valence was higher for positive words than for all other word types, while negative and taboo words were rated lower on valence than neutral and positive words. It is worth noting that the standard deviation for taboo words was higher than for other word types; although valence ratings for taboo words were on average lower than for neutral words, some taboo words (e.g., *erection, erotic*) received ratings around or slightly above the midpoint of the valence scale. Arousal ratings were highest for taboo words. Moreover, arousal ratings for high arousal positive and high arousal negative words (as selected from Moors et al, 2013) were higher than those for low arousal positive, low arousal negative, and neutral words. As expected, taboo words were rated higher than all other word types on general taboo, personal taboo, and insult. Note that positive words, whether high or low arousal, and neutral words scored near the bottom ends of the general taboo, personal taboo and insult rating scales.

**Table 3:** Mean ratings and standard deviations (in parentheses) for each word type, positive and negative words broken down by arousal level (high and low)

Word type	Valence	Arousal	General Taboo	Personal Taboo	Insult
Taboo	2.79 (0.95)	3.87 (0.59)	3.59 (1.05)	2.83 (0.98)	3.13 (1.53)
Positive					
High arousal	5.59 (0.27)	3.44 (0.27)	1.13 (0.17)	1.06 (0.09)	1.02 (0.02)
Low arousal	4.87 (0.36)	2.31 (0.28)	1.08 (0.08)	1.04 (0.04)	1.04 (0.07)
Negative					
High arousal	2.43 (0.30)	3.65 (0.32)	2.31 (0.58)	1.96 (0.44)	1.77 (0.55)
Low arousal	2.91 (0.39)	2.55 (0.39)	1.76 (0.44)	1.53 (0.28)	1.81 (0.49)
Neutral	4.14 (0.22)	2.39 (0.25)	1.05 (0.08)	1.03 (0.05)	1.07 (0.17)

### Associations between rating variables

We analyzed associations between rating variables using a regression analysis. We tested both linear and quadratic relations for each analysis. The linear model was always entered first in the regression analyses (mean scores of the predictor variable) and the quadratic model was always entered second (squared mean scores of the predictor variable). We chose to test quadratic relations for every analysis because earlier studies (e.g., Janschewitz, 2008; Moors et al., 2013;) have shown that valence and arousal have a non-linear relation. With the exception of the quadratic relation between valence and arousal, we did not have prior expectations regarding these analyses.

#### Valence and arousal

**Fig. 3:**
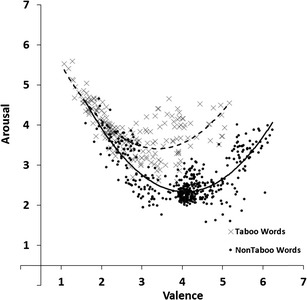
Mean arousal ratings plotted against the mean valence ratings for all 672 words. Lower scores indicate more passive arousal or negative valence ratings, higher scores indicate more active arousal or positive valence ratings

To estimate the associations between the valence and arousal dimensions we carried out a regression analysis with arousal ratings as the response variable and valence ratings as the predictor variable. Figure 3 shows the scatterplot of mean arousal ratings plotted against the mean valence ratings for all 672 words. Each dot represents one of the 672 words, the solid line shows the relation between valence and arousal for non-taboo words and the dotted line shows the relation for taboo words. The linear relation accounted for 25% of the variance (*R* ^*2*^ = .25, *F*(1, 670) = 226.34, *p* < .001), the quadratic relation accounted for 58% of the variance (*R* ^*2*^_*change*_ = .33, *F*_*change*_(1, 669) = 513.63, *p* < .001). This shows that words rated low or high on valence were on average rated higher on arousal than words that received ratings around the midpoint of the valence scale.

As exploratory analyses, we carried out separate regression analyses for both taboo and non-taboo words, again with arousal ratings as the response variable and valence ratings as the predictor variable. For non-taboo words, the linear relation accounted for 3% of the variance (*R* ^*2*^ = .03, *F*(1, 468) = 13.28, *p* < .001), the quadratic relation accounted for 64% of the variance (*R* ^*2*^_*change*_ = .61, *F*_*change*_(1, 467) = 789.52, *p* < .001). For taboo words, the linear relation accounted for 20% of the variance (*R* ^*2*^ = .20, *F*(1, 200) = 49.02, *p* < .001), the quadratic relation accounted for 57% of the variance (*R* ^*2*^_*change*_ = .37, *F*_*change*_(1, 199) = 173.52, *p* < .001).

A closer look at Fig. 3 shows that most words with a mean valence rating around the midpoint had mean arousal ratings below 3.00. Of all 371 words with a mean valence rating higher than 3.00 and lower than 5.00 only 68 words (18%) had a mean arousal rating higher than 3.00. Of those 68 words, 62 words (91%) were taboo words and six were non-taboo words. Of the 303 words with a mean arousal rating of 3.00 or lower only ten words (3%) were taboo words and 293 words were non-taboo words. Thus, there seems to be a clear separation between taboo and non-taboo words. Almost all medium valence words have relatively low arousal ratings with the exception of taboo words.

To summarize, we found a quadric relation between our valence and arousal ratings. This U-shaped pattern has also been found in other rating studies (e.g., Bradley & Lang, 1999; Janschewitz, 2008; Monnier & Syssau, 2014; Moors et al., 2013). Here we show that the quadratic relation also holds for taboo words. This is a finding that, to the best of our knowledge, has not been reported before.

#### General and personal taboo

**Fig. 4:**
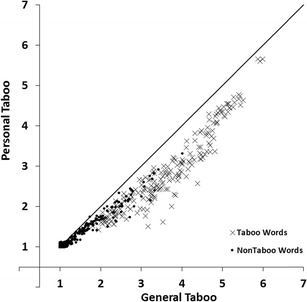
Mean personal taboo ratings plotted against the mean general taboo ratings for all 672 words. Lower scores indicate not/low taboo, higher scores indicate more taboo

To estimate the associations between the general taboo and personal taboo ratings we carried out a regression analysis with personal taboo ratings as the response variable and general taboo ratings as the predictor variable. Figure 4 shows the scatterplot of mean personal taboo ratings plotted against the mean general taboo ratings for all 672 words. The linear relation accounted for 96% of the variance (*R* ^*2*^ = .96, *F*(1, 670) = 14682.95, *p* < .001), the quadratic relation accounted for 97% of the variance (*R* ^*2*^_*change*_ = .01, *F*_*change*_(1, 669) = 282.49, *p* < .001). Words rated high on general taboo were also rated high on personal taboo. Although the correlation between personal taboo and general taboo ratings was very high, personal taboo ratings were on average somewhat lower than general taboo ratings. This is evident in Fig. 4 where the large majority of the words are located below the diagonal (see also Table 3).

Because of the very high correlation between general and personal taboo ratings we only discuss regression analyses that involve general taboo ratings in the remainder of the paper. The correlation between general taboo ratings and another variable will necessarily be very similar in magnitude and direction to the correlation between personal taboo ratings and that variable.

#### Taboo and insult

**Fig. 5:**
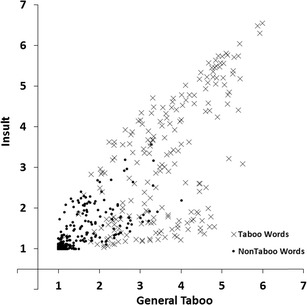
Mean insult ratings plotted against the mean general taboo ratings for all 672 words. Lower scores indicate not/low insulting or not/low taboo, higher scores indicate more insulting or taboo

To estimate the associations between the general taboo and insult dimensions we carried out a regression analysis with insult ratings as the response variable and general taboo ratings as the predictor variable. Figure 5 shows the scatterplot of mean insult ratings plotted against the mean general taboo ratings for all 672 words. The linear relation accounted for 68% of the variance (*R* ^*2*^ = .68, *F*(1, 670) = 1435.03, *p* < .001, the quadratic relation accounted for 69% of the variance (*R* ^*2*^_*change*_ = .01, *F*_*change*_(1, 669) =20.76, *p* < .001). Words rated high on general taboo also tended to be rated high on insult. However, as can be seen in Fig. 5, this is not the case for all words. Some words have a high taboo rating but a low insult rating. Taboo words are therefore not necessarily insulting. Non-insulting taboo words mainly include sexual terms (e.g., *erection, orgasm,* and *sperm*). These words are rarely used as insults but are considered taboo words.

#### Taboo and valence

**Fig. 6:**
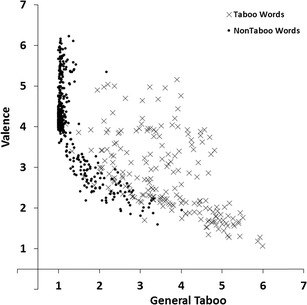
Mean valence ratings plotted against the mean general taboo ratings for all 672 words. Lower scores indicate not/low taboo or negative valence, higher scores indicate more taboo or positive valence

To estimate the associations between the general taboo and valence dimensions we carried out a regression analysis with valence ratings as the response variable and general taboo ratings as the predictor variable. Figure 6 shows the scatterplot of mean valence taboo ratings plotted against the mean general taboo ratings for all 672 words. The linear relation accounted for 51% of the variance (*R* ^*2*^ = .51, *F*(1, 670) = 705.93, *p* < .001), the quadratic relation accounted for 55% of the variance (*R* ^*2*^_*change*_ = .04, *F*_*change*_(1, 669) = 50.27, *p* < .001). Words rated high on general taboo were generally rated low on valence, but there were some exceptions. Positive valence taboo words mainly include sexual terms.

#### Insult and valence

**Fig. 7:**
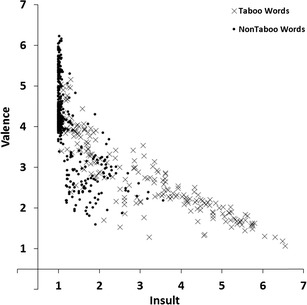
Mean valence ratings plotted against the mean insult ratings for all 672 words. Lower scores indicate not/low insulting or negative valence, higher scores indicate more insulting or positive valence

To estimate the associations between the insult and valence dimensions we carried out a regression analysis with valence ratings as the response variable and insult ratings as the predictor variable. Figure 7 shows the scatterplot of mean valence ratings plotted against the mean insult ratings for all 672 words. The linear relation accounted for 56% of the variance (*R* ^*2*^ = .56, *F*(1, 670) = 859.39, *p* < .001), the quadratic relation accounted for 63% of the variance (*R* ^*2*^_*change*_ = .07, *F*_*change*_(1, 669) =131.23, *p* < .001). Words that were rated high on insult were rated low on valence.

#### Taboo and arousal

**Fig. 8:**
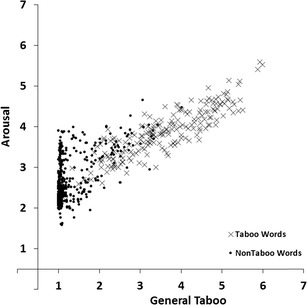
Mean general taboo ratings plotted against the mean arousal ratings for all 672 words. Lower scores indicate not/low taboo or passive arousal, higher scores indicate more taboo or active arousal

To estimate the associations between the general taboo and arousal dimensions we carried out a regression analysis with arousal ratings as the response variable and general taboo ratings as the predictor variable. Figure 8 shows the scatterplot of mean arousal ratings plotted against the mean general taboo ratings for all 672 words. The linear relation accounted for 71% of the variance (*R* ^*2*^ = .71, *F*(1, 670) = 1673.95, *p* < .001), the quadratic relation accounted for 71% of the variance (*R* ^*2*^_*change*_ < .01, *F*_*change*_(1, 669) = 7.23, *p* = .007). Words that were rated high on taboo were rated high on arousal.

#### Insult and arousal

**Fig. 9:**
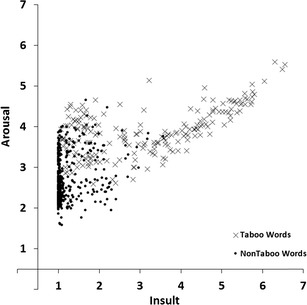
Mean arousal ratings plotted against the mean insult ratings for all 672 words. Lower scores indicate not/low insulting or passive arousal, higher scores indicate more insulting or active arousal

To estimate the associations between the insult and arousal dimensions we carried out a regression analysis with arousal ratings as the response variable and insult ratings as the predictor variable. Figure 9 shows the scatterplot of mean arousal ratings plotted against the mean insult ratings for all 672 words. The linear relation accounted for 45% of the variance (*R* ^*2*^ = .45, *F*(1, 670) = 551.54, *p* < .001), the quadratic relation accounted for 45% of the variance (*R* ^*2*^_*change*_ < .01, *F*_*change*_(1, 669) = 1.46, *p* = .228). Words that were rated high on insult were rated high on arousal.

### Gender differences

In order to analyze gender differences, we performed correlational analyses on all variables between females and males. Thirty-two females and 12 males rated words on arousal. Twenty-six females and 17 males rated words on valence.

In order to calculate these correlations, we calculated the mean rating for each word per gender. Overall, this analysis shows high agreement between male and female participants on all variables: for arousal, *r* = .92, valence, *r* = .97, general taboo, *r* = .97, personal taboo, *r* = .96, insult, *r* = 98.

Table 4 shows the means and standard deviations of taboo words for men and women per rating variable. There were slight gender differences. Women rated taboo words as more taboo and insulting than men did and men rated taboo words as more positive and more arousing than women did (all *p*s < .01).

**Table 4:** Mean ratings and standard deviations (in parentheses) of taboo words for men and women

Gender	Valence	Arousal	General taboo	Personal taboo	Insult
Men	2.98 (0.95)	4.48 (0.63)	3.37 (1.10)	2.52 (0.89)	2.85 (1.38)
Women	2.66 (0.97)	3.69 (0.60)	3.71 (1.04)	2.99 (1.04)	3.26 (1.60)

### Religiosity

In order to analyze religiosity, we performed correlational analyses on all variables between religious and nonreligious participants. For the valence ratings, ten religious students participated and for the arousal ratings 8 religious students participated (for a total of 18 religious participants). Of these 18 students, 12 identified themselves as Christian, one as Islamic, three as Hindu, one as Jehovah’s witness, and one described her religion as “non-specific.” Of the 18 religious participants, six participants indicated that they visited their house of worship every week, one participant indicated she visited her house of worship between once a week and once a month, six participants indicated they visit their house of worship less than once a month, and five participants indicated that they never visit their house of worship. In addition, four nonreligious participants indicated that they visited a house of worship a few times per year (i.e., “less than once a month,” but not “never”). Because of the small numbers of participants, we did not use information regarding the frequency of visits to their house of worship in our analyses. Rather, we divided the participants based on the question that asked participants whether they were religious or not.

In order to calculate correlations on word ratings between religious and nonreligious participants, we calculated the mean ratings for each word for religious and nonreligious participants. Overall, these analyses showed high agreement between groups on all variables; for arousal, *r* = .96, valence, *r* = .92, taboo general, *r* = .98, taboo personal, *r* = .96, insult, *r* = 97.

Table 5 shows the means and standard deviations of taboo words for religious and nonreligious participants per rating variable. There were slight differences between religious and nonreligious participants. Religious participants rated taboo words higher on personal taboo and as more insulting than nonreligious participants did (*p*s < .01). Thus interestingly, whereas religious and nonreligious participants rated taboo words differently on persnal taboo they did not rate these words different on general taboo; a mixed ANOVA with rating (personal taboo vs. general taboo as a within-subjects factor and religiosity as a between subjects factor revealed a significant interaction, *F*(1, 402) = 296.05, *p* < .001. The sample sizes for valence and arousal ratings were very small for religious participants. Consequently, no strong conclusions for the absence of an effect of religiosity on these ratings should be drawn.

**Table 5:** Mean ratings and standard deviations (in parentheses) of taboo words for religious and nonreligious participants

Gender	Valence	Arousal	General taboo	Personal taboo	Insult
Religious	2.57 (0.89)	4.22 (0.69)	3.69 (1.08)	3.41 (1.14)	3.59 (1.54)
Nonreligious	2.85 (0.98)	3.80 (0.58)	3.57 (1.06)	2.68 (0.95)	3.01 (1.53)

## Discussion

The purpose of this study was to provide researchers with taboo norms for a number of Dutch words. We had psychology students rate 672 Dutch words on valence, arousal, general tabooness, personal tabooness, and insult. We included a number of different word types: taboo words, positive words, negative words, and neutral words. All ratings were given on a 7-point Likert scale. We compiled these ratings in the Dutch Taboo Norms (DTN). Our ratings should be useful to researchers using Dutch-speaking subject populations who want to use taboo words in their study.

We found a number of interesting patterns in our norm data. First, the scatter plot for valence and arousal ratings (Fig. 3) showed that words that were rated high or low on valence (i.e., words with high valence extremity) tended to be rated high on arousal (relative to words with a neutral valence rating; Bradley & Lang, 1999; Janschewitz, 2008; Monnier & Syssau, 2014; Moors et al., 2013). Here we show for the first time that he quadratic relation between valence and arousal holds when only taboo words are considered. Second, correlations between taboo and insult ratings indicated that words that had a high taboo rating showed a high insult rating (Fig. 5). This indicates that on average words that were highly taboo were more insulting. However, this is not the case for all words. Several words are considered taboo (e.g., sexual terms like *erection, orgasm,* and *sperm*) but are rarely used as insults. Third, the scatterplot for taboo and valence ratings (Fig. 6) and the scatterplot for insult and valence ratings (Fig. 7) showed that words rated high on taboo and words rated high on insult tended to be rated low on valence. However, whereas all words with a mean rating of 2 or higher on insult were rated low on valence (i.e., <4.0, below the midpoint of the scale), this was not the case for words with a mean rating of 2 or higher on general taboo. Nineteen words with a mean rating of 2 or higher on general taboo received a mean valence rating above the midpoint of the scale. Finally, we found that words rated high on taboo and words rated high on insult also tended to be rated high on arousal (Figs. 8 and 9).

We obtained high split-half reliabilities within our sample for the arousal, valence, general taboo, personal taboo, and insult ratings. We found high correlations between our newly collected data and norms for valence (*r* = .98) and arousal (*r* = .77) collected by Moors et al. (2013). Janschewitz (2008) correlated her ratings with those of the Affective Norms for English Words (ANEW; Bradley & Lang, 1999) database and, like us, found higher correlations for valence (*r* = .94) than for arousal (*r* = .72). This may reflect inherently lower person-to-person consistency for arousal than for valence. It may also reflect a larger influence of list composition on arousal ratings than on valence ratings. That is, the nature (and number) of other words on the list may affect arousal ratings, more so than it affects valence ratings.

In addition to the lower correlations for arousal words between databases, mean arousal ratings were much lower in our study than in Moors et al. (2013), as shown in Table 6. Similarly, Janschewitz (2008) obtained lower arousal ratings for a set of words that was also rated for the ANEW database (Bradley & Lang, 1999). In both our study and in the Janschewitz study, participants rated a large number of taboo words, which may have caused participants to become less aroused by the words. That is, the same word might be rated higher on arousal when presented in a context with a low number of taboo words than in a context with a high number of taboo words. Another possibility is that explicit instructions to rate words on tabooness dampens some of the surprise and embarrassment, causing arousal ratings to become lower. Future research could focus on the effects of the presence of a high percentage of taboo words in a rating study and the effects of rating the tabooness of words in addition to arousal.

**Table 6:** Mean arousal ratings and standard deviations (in parentheses) for each word type for the DTN, Moors et al. (2013), Janschewitz (2008), and ANEW

	DTN	Moors et al.	Janschewitz	ANEW
Taboo	3.87 (0.59)	4.65 (0.59)	4.39 (0.93)	5.47 (0.99)
Positive	2.88 (0.63)	4.26 (1.29)	2.92 (0.79)	5.39 (1.07)
Negative	3.10 (0.66)	4.07 (1.54)	2.89 (0.62)	5.56 (1.15)
Neutral	2.39 (0.25)	3.95 (0.77)	1.66 (0.31)	3.91 (0.53)

*Note*. DTN and Moors et al. ratings were rated on a 7-point Likert scale. Janschewitz (2008) and ANEW ratings were rated on a 9-point scale. Ratings for DTN and Moors et al. (2013) are based on the same Dutch words. Ratings for Janschewitz and ANEW are based on the same English words

General and personal taboo ratings were highly correlated (see Fig. 4), more so than any other variables in our norming study. One could argue that participants did not make a clear distinction between how taboo they believe the word is in general or to them personally. However, only one participant mentioned that she did not clearly understand the distinction between general and personal taboo and had rated both the same. Although general and personal taboo ratings were very highly correlated, they were not identical; personal taboo ratings were on average lower than general taboo ratings. Janschewitz (2008) also reported this finding for the comparison of (personal) offensiveness ratings and (general) tabooness ratings. This suggests that personal reactions towards taboo words are less extreme than what participants perceive these reactions to be in general.

Taboo ratings for men and women were highly correlated. Although these correlations were high, women rated taboo words as more taboo and insulting than men did and men rated taboo words as more positive and more arousing than women did (see Table 4). This difference seems to be present for most taboo words regardless of which type of taboo words rated. Taboo ratings for religious and nonreligious participants were highly correlated. Nevertheless, religious participants showed higher personal taboo and insult ratings than nonreligious participants did (see Table 5). It seems that religious people might be more offended by taboo words than nonreligious people. Specifically, religious and nonreligious people do not seem to differ in their general idea how taboo words are but religious people are more personally offended by taboo words than non-religious people. It remains to be seen whether difference in gender and religiosity mediate the effect of taboo words on cognitive performance and physiological responses.

A number of non-taboo words had a relatively high taboo rating. These words were mostly low valence - high arousal words related to violence, such as *murder, abuse, slaughter,* and *war*. We would like to stress that taboo and non-taboo words were chosen according to the experimenters’ discretion prior to data collection. This implies that we did not know if those words would be rated high or low on the taboo scales. It is clear that certain highly negative and arousing words were considered taboo by some participants. Thus although taboo ratings overall agreed well with our intuitions and those of other researchers, there were some “taboo” words with relatively low taboo ratings and “non-taboo” words with relatively high taboo ratings.

As can be seen in Table 3, our mean general taboo, personal taboo, and insult ratings were all below the midpoint of the rating scale (i.e., below 4). As a reviewer pointed out, several factors may have contributed to this finding. First, it could be that participants perceived themselves as “cool” and did not want to be perceived as being easily upset. Second, habituation may have played a role. Participants are possibly exposed to a large number of “taboo” words or events in their daily life and are simply not that offended by them anymore. The large number of taboo words that were presented in the study may have caused some habituation. Third, our selection of taboo words also contained taboo words that are taboo in certain context but not in others (e.g., *“nicht”* can mean *cousin* or *fag*). Some words were ambiguous and consequently participants may have accessed the non-taboo meaning of the word, which resulted in a low taboo score for that word.

With the DTN we have attempted to capture a large corpus of words that might be considered taboo to a sample of psychology students. We do not claim that the DTN is exhaustive. For example, one reviewer pointed out that the Dutch equivalent of asshole *“klootzak”* was (accidentally) not included in our sample. Although our sample does not include all possible taboo words, we are convinced that the DTN database is a useful tool for researchers interested in the effects of taboo words on cognitive processing.

The sample used for this study consisted of psychology students at the Erasmus University Rotterdam (rather than a random sample from the population at large). This should be considered when using the DTN. Moors et al. (2013) took samples from several Dutch and Belgian universities and concluded that their ratings did not show strong regional differences (apparent in a high correlation between samples from different regions). We also found high correlations for valence and arousal ratings for the words that were included in the present study and that of Moors et al. This suggests that our data will be useful to researchers wishing to test Dutch-speaking students more generally. Nevertheless, the tabooness of some words might be noticeably different for students at Dutch and Belgian universities. Thus, some caution should be exercised when using these norms in Belgium.

Whether affective norms collected with students are also useful for studies with nonstudent populations is not certain. Factors such as age, education, socio-economic status, religion and gender may influence affective ratings in general and perhaps even stronger for taboo words. We did find differences in taboo and insult ratings between religious and nonreligious students as well as between male and female students. Nevertheless, ratings of religious and nonreligious students and male and female students were overall highly correlated. This suggests that our taboo ratings may be of value beyond the population from which they were sampled.

## Footnotes

1 Originally, we tested 81 participants. Because our original sample contained only 13 males, we collected data from 19 additional males for the revised manuscript.

2 In Dutch, compounds are written as a single word, even when more than two words are combined (e.g., *langeafstandsloper* [*long-distance runner*]). Diseases are often used as swear words (although not all diseases are used as such) on their own and in combination with other (swear) words.

3 Due to participant dropout, not all files have been rated. Original file numbers can be found in the “Raw Participant Data” file in the Online Supplementary Material.

4 Data from the context variable will not be discussed because there was relatively little variation in the answers. Participants mostly wrote down “*always,” “when used as a swear word/as an abusive word,”* “*when in the company of parents/elders,*” or left it blank.
